# Understanding Gratification Disorder in Childhood: A Comprehensive Review of Diagnosis, Differential Diagnosis, and Management Approaches

**DOI:** 10.7759/cureus.70415

**Published:** 2024-09-28

**Authors:** Imyarila Longkumer, Ragini Patil, Ateeba Ahmed

**Affiliations:** 1 Psychiatry, Jawaharlal Nehru Medical College, Datta Meghe Institute of Higher Education and Research, Wardha, IND

**Keywords:** behavioral interventions, childhood disorders, developmental outcomes, diagnostic criteria, gratification disorder, impulse control

## Abstract

Gratification disorder (GD) in childhood is characterized by difficulty delaying immediate rewards and controlling impulsive behaviors. This disorder manifests as a persistent struggle to wait for longer-term rewards and a tendency toward impulsive decision-making, which can disrupt academic performance, social interactions, and daily functioning. The relevance of GD is highlighted by its potential to impede the development of crucial skills such as self-control, problem-solving, and social competence. This comprehensive review aims to provide an in-depth understanding of GD by examining its diagnostic criteria, exploring differential diagnoses, and evaluating various management strategies. Key objectives include clarifying the characteristics of GD, distinguishing it from other disorders with overlapping symptoms, and assessing effective interventions, including behavioral therapies, pharmacological options, and educational modifications. The review underscores the importance of accurate diagnosis and tailored interventions for clinicians, educators, and parents, emphasizing the need for a multidisciplinary approach to support affected children. Understanding GD is essential for improving developmental outcomes and ensuring that children receive appropriate support to navigate the challenges associated with the disorder. Continued research and advancements in diagnostic and therapeutic practices are crucial for enhancing the management of GD and fostering better long-term outcomes for affected individuals.

## Introduction and background

Gratification disorder (GD) is characterized by difficulty delaying immediate rewards or controlling impulsive behaviors in children [1]. This condition is marked by a persistent struggle to wait for long-term rewards and an overwhelming desire for instant gratification. Children with GD may frequently exhibit impulsive decision-making, impatience, and a preference for immediate rewards over delayed but more substantial ones [2]. These behaviors are not just occasional lapses but represent a significant and persistent challenge that affects the child’s ability to plan, focus, and engage in goal-directed activities [3]. The relevance of GD in childhood is underscored by its potential impact on multiple developmental domains. Children grappling with GD may face difficulties in academic settings, struggle with forming and maintaining interpersonal relationships, and encounter disruptions in their daily routines [4]. The disorder can impede the development of essential skills such as self-control, problem-solving, and effective social interactions. As such, GD can significantly hinder a child's overall adaptive functioning and long-term success, making it a critical area of concern for both parents and professionals [4].

The primary objective of this review is to provide a comprehensive understanding of GD. This involves thoroughly examining the disorder's characteristics, including its diagnostic criteria and typical behavioral and psychological presentations. By exploring the current frameworks used to diagnose GD, the review aims to clarify how this disorder is identified and differentiated from other conditions with overlapping symptoms. Additionally, the review evaluates various management strategies, including behavioral therapies, pharmacological interventions, and educational modifications, to present a well-rounded approach to managing GD in children.

Understanding GD is crucial for several key stakeholders. For clinicians, accurate diagnosis and effective management are vital to improving a child's developmental trajectory and quality of life. Recognizing GD early allows clinicians to implement targeted interventions that address the disorder’s impact on academic performance, social interactions, and emotional well-being [5]. For educators, a clear understanding of GD facilitates the design of supportive learning environments that accommodate the unique needs of affected students, enhancing their academic and social experiences. Finally, for parents, knowledge of GD enables them to adopt effective home-based strategies and collaborate more effectively with professionals. This comprehensive understanding ultimately supports better developmental outcomes for children with GD and promotes more informed and effective interventions across various settings [6].

## Review

Clinical presentation of GD

GD, commonly observed in children between the ages of three months and three years, is characterized by a variety of self-stimulatory behaviors that may resemble seizures or other movement disorders [1]. Notable symptoms include stereotyped episodes lasting from five to 40 minutes, during which the child may exhibit rhythmic movements and vocalizations such as quiet grunting, facial flushing, and sweating [7]. During these episodes, children may apply pressure to the perineum and adopt specific postures, such as crossing or flexing their legs. Importantly, children remain conscious throughout these episodes and can often be distracted from the behavior [7]. In the home environment, parents might observe their child rubbing their genitals against objects or their own hands, sometimes appearing fixated or dazed during these moments. In educational settings, the stereotyped movements and vocalizations can become disruptive, raising concerns among teachers and peers. If unaddressed, the frequency and duration of these episodes may increase over time, leading to further complications in the child’s daily life [8].

**Figure 1:**
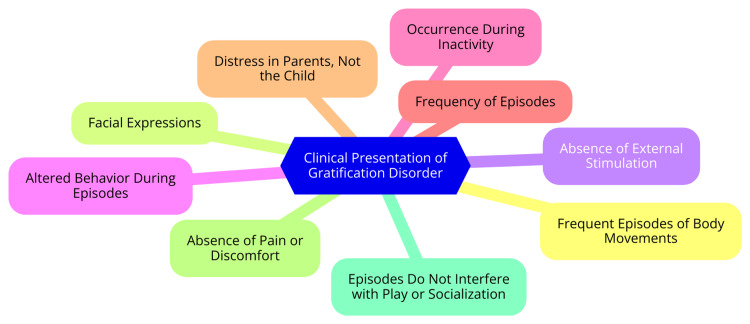
Clinical presentations of GD GD, gratification disorder Image Credit: Dr Imyarila Longkumer

Although GD is generally considered a benign behavioral variant in early childhood, the repetitive nature of these self-stimulatory behaviors can impact cognitive, emotional, and social development [1]. Children with GD may struggle with impulse control and exhibit deficits in executive functioning, which can impede their ability to delay gratification and resist the urge to engage in self-stimulatory behaviors. This lack of self-regulation can affect their focus and learning capabilities in school, potentially influencing academic performance [1]. Emotionally, children may experience feelings of shame or guilt if their behaviors are met with scolding or punishment from caregivers. Such negative reinforcement can adversely impact their self-esteem and overall emotional well-being. Socially, the disruptive nature of these behaviors in educational settings may lead to peer rejection or bullying, further isolating the child. It is crucial for parents and caregivers to recognize GD as normal behavior and to offer appropriate guidance and support [9]. Professional help is recommended if the behaviors become excessive or cause significant distress. Children with GD can develop healthy impulse control and executive functioning skills with proper management, enabling them to navigate their emotional and social environments more effectively [10]. Clinical presentations of GD are illustrated in Figure 1.

Diagnostic criteria

GD, often referred to as "benign idiopathic infantile dyskinesia," lacks specific diagnostic criteria in the DSM-5 and ICD-10/ICD-11 [1]. This condition is generally considered a variant of normal behavior in young children rather than a formal psychiatric disorder. The behaviors associated with GD are typically characterized by self-stimulatory actions that do not align neatly with existing categories of mental health conditions [1]. While the ICD-11 acknowledges various movement disorders and behavioral issues, it does not offer a distinct classification for GD. Instead, it emphasizes the importance of thorough clinical assessment to differentiate these behaviors from other conditions, such as epilepsy, paroxysmal movement disorders, and gastrointestinal issues, which may be misdiagnosed due to overlapping symptoms [11]. Although formal criteria are absent, clinicians often use specific features to identify GD. Key indicators include the age of onset, typically observed in children aged three months to three years, with a potential second peak during adolescence. Behavioral characteristics involve episodes of self-stimulatory behaviors that often occur when the child is alone or bored [5]. These episodes may feature stereotyped movements, vocalizations (such as grunting), facial flushing, and characteristic posturing of the lower extremities. Importantly, these episodes occur without altered consciousness and can be interrupted by distraction or engagement in other activities. A normal physical examination and laboratory results further support the diagnosis [5].

Currently, no standardized tests or questionnaires are specifically designed for diagnosing GD. However, clinicians may use general developmental screening tools like the Ages and Stages Questionnaire or the Denver Developmental Screening Test to assess overall child development and exclude other developmental concerns. These tools provide a broader context for understanding the child's behavior and development [12]. Clinical interviews with parents are essential for obtaining a comprehensive child behavior history. During these interviews, healthcare providers can explore the frequency, duration, and context of self-stimulatory behaviors and any associated factors, such as stress or environmental triggers. Observational methods, including video recordings of episodes, are particularly valuable for accurate diagnosis [13]. These recordings assist in distinguishing gratification behaviors from other conditions, such as seizures or movement disorders, which parents may find challenging to describe accurately. By combining clinical interviews and observational methods, clinicians can achieve a more accurate and nuanced understanding of the child's behavior, leading to appropriate management and support [5]. Comprehensive diagnostic criteria for GD in childhood are detailed in Table 1.

**Table 1:** Comprehensive diagnostic criteria for GD in childhood GD, gratification disorder

Criterion	Detailed Description
Age of Onset [5]	GD generally manifests between 3 months and 3 years of age. It is most commonly observed in toddlers and early childhood. Symptoms may emerge earlier but often go unnoticed due to the subtlety of initial behaviors.
Behavioral Manifestations [14]	The child engages in repetitive, stereotypical rhythmic movements such as crossing legs tightly, rocking, or applying pressure to the pelvic region. These behaviors are often mistaken for abdominal discomfort or other conditions. Movements may also include grinding the teeth, facial flushing, or sweating. The behavior tends to occur during periods of boredom, inactivity, or while seated (e.g., in a car seat, high chair, or during screen time).
Response to Distraction [1]	One of the distinguishing features of GD is that the child can often be distracted from the behavior through intervention or engagement in a stimulating activity. For instance, a caregiver may stop the behavior by offering a toy, changing the environment, or providing a new focus of attention. This contrasts with more pathological movements, which are difficult to interrupt.
Absence of Associated Symptoms [15]	No neurological or physical symptoms, such as loss of consciousness, confusion, or involuntary seizure-like activity. The absence of these symptoms helps differentiate GDs from seizure disorders like epilepsy. The child remains alert and responsive throughout the episode.
Differentiation from Seizures [16]	Unlike seizures, children with GD maintain full consciousness, awareness, and interaction with the environment. The movements are deliberate and goal-directed rather than involuntary. There is also no postictal phase (confusion or fatigue following a seizure). A medical history or observation of the episodes can help confirm this distinction.
Non-distressful Nature [17]	The behaviors exhibited during GD episodes are generally non-painful and not associated with distress or discomfort. The child may appear flushed or focused but does not seem distressed. In contrast, other self-soothing behaviors in children, such as thumb sucking, may indicate stress or emotional upset.
Environmental Context [5]	GD episodes are more likely to occur in specific settings, particularly during quiet or unstimulating moments, such as long car rides, naps, or watching TV. It is rarely observed in highly stimulating environments (e.g., playgrounds and social gatherings). This context helps distinguish it from other movement disorders or seizures, which are less context-dependent.
No Developmental Impact [18]	Unlike many neurodevelopmental or behavioral disorders, GD does not interfere with a child’s cognitive, social, or motor development. Children with this disorder achieve normal developmental milestones appropriate for their age group. There is no regression in skills, and the disorder usually resolves independently without intervention as the child matures.
Frequency and Duration of Episodes [19]	The frequency of the behaviors varies but may be more common during extended periods of inactivity. Each episode lasts a few to several minutes, and behaviors are often self-limited. Caregivers typically report that the child can stop the behavior, especially with distraction or environmental change.
Parental Observation [20]	Parents often report feeling confused or concerned when they first notice the behavior, mistaking it for pain, constipation, or abdominal distress. However, they eventually notice that the behavior is not accompanied by crying or discomfort, leading to suspicion of gratification behavior. Medical evaluation is often sought for reassurance.

Differential diagnosis

GD is often misdiagnosed due to its overlapping symptoms with various common childhood conditions. Understanding these overlaps and differentiation methodologies is crucial for accurate diagnosis and management [5]. Common conditions with symptoms similar to GD include attention-deficit/hyperactivity disorder (ADHD), oppositional defiant disorder (ODD), and autism spectrum disorder (ASD). Children with ADHD may display impulsive behaviors and difficulty maintaining attention, which can sometimes appear as repetitive or self-stimulatory actions [21]. However, the context and nature of these behaviors differ from those seen in GD. ODD is characterized by angry or irritable mood, argumentative behavior, or vindictiveness. While children with ODD may exhibit disruptive behaviors, these are typically more related to defiance rather than self-stimulation, distinguishing them from GD. ASD can also include repetitive behaviors and restricted interests that may superficially resemble GD's self-stimulatory actions [22]. However, the motivations and contexts of these behaviors in ASD often involve social communication challenges, distinguishing them from GD. Accurate differentiation of GD from other disorders requires a thorough clinical assessment. Key features of GD include its typical onset between three months and three years and specific behavioral characteristics [23].

GD episodes are often stereotyped, involving vocalizations, facial flushing, and perineal pressure, without loss of consciousness. Importantly, these episodes can be interrupted by distraction, a feature not typical in seizures or other movement disorders. Additionally, GD episodes are usually brief and may occur multiple times a day, often linked to boredom or lack of stimulation [23]. Comprehensive evaluations, including detailed histories and physical examinations, are crucial for accurate diagnosis [24]. Video recordings of episodes can provide invaluable insights, helping to differentiate GD from conditions like epilepsy or movement disorders, which may present similarly but have distinct characteristics. Multi-disciplinary assessments involving pediatricians, neurologists, and psychologists can enhance diagnostic accuracy by integrating various perspectives and expertise, ensuring that all potential underlying issues are considered [24]. Accurate diagnosis of GD is essential to avoid unnecessary investigations and treatments, such as anti-epileptic medications, which may be initiated due to misdiagnosis. Understanding the unique presentation of GD and employing effective differentiation methodologies can significantly improve clinical outcomes for affected children and their families [1]. Differential diagnosis of GD in childhood is detailed in Table 2.

**Table 2:** Differential diagnosis of GD in childhood OCD, obsessive-compulsive disorder; ASD, autism spectrum disorder; GD, gratification disorder

Condition	Key Features	Differentiating Factors From GD
Epilepsy (Focal Seizures) [25]	Repetitive movements or posturing may occur. Seizures may be subtle and involve only a part of the body (e.g., head, arms, or legs). Consciousness can be altered or lost.	Seizures often involve a lack of awareness or altered consciousness. Postictal confusion or lethargy follows the seizure. The child cannot be easily distracted from the episode.
ASD [26]	Repetitive behaviors and self-stimulatory actions (e.g., hand flapping, rocking, or spinning) are common. Difficulty with social interactions and communication deficits may be present.	Unlike GD, ASD is associated with delayed social and communication skills, and repetitive behaviors are often persistent and not situational. These behaviors are not typically linked to specific times of inactivity.
OCD [27]	Involves intrusive thoughts (obsessions) and repetitive actions (compulsions) to reduce anxiety. Compulsive actions are often ritualistic and performed in response to distressing thoughts.	OCD compulsions are driven by anxiety and are often aimed at reducing obsessive thoughts. Children with OCD may express distress or fear, whereas GD is non-anxiety-driven.
Stereotypic Movement Disorder [28]	Repetitive, nonfunctional motor behavior (e.g., hand-waving and head-banging) that interferes with normal activities. Movements are involuntary and repetitive.	Stereotypies are generally involuntary, non-goal-oriented, and persist despite attempts to distract the child. In contrast, GD is voluntary and can be interrupted.
Infantile Spasms [29]	Sudden jerking movements of the head, arms, and legs. Often occurs in clusters and is associated with developmental delay. These episodes are typically brief.	Sudden, involuntary movements with no clear goal or purpose characterize infantile spasms. They are usually associated with developmental delays and do not respond to distraction.
Gastrointestinal Disorders (e.g., Constipation) [30]	Children with constipation may show signs of discomfort, including straining, crying, or holding the abdomen. This can be mistaken for the physical posturing seen in GD.	Constipation typically involves pain or discomfort; the child may cry or appear distressed. Unlike GD, gastrointestinal symptoms are usually linked to bowel movements.
Tourette Syndrome [31]	Characterized by involuntary tics, including motor and vocal tics. Movements may be sudden, repetitive, and brief. Vocal tics may accompany motor tics.	Tics in Tourette Syndrome are involuntary and can involve vocalizations. GD behaviors are goal-directed and can be interrupted with distraction, unlike tics.
Sexual Abuse [32]	Behavioral changes include unusual sexualized behavior, inappropriate knowledge of sexual acts, or fear of specific individuals or places.	Sexual abuse may manifest in sexualized behavior but is typically accompanied by emotional or behavioral changes, such as withdrawal, fear, or anxiety. A full psychosocial assessment is required to differentiate from GD.
Self-Soothing Behaviors [33]	Common in young children and may include thumb-sucking, hair-pulling, or rocking to comfort them during stress or sleep. Often seen in toddlers.	Self-soothing behaviors, while repetitive, are generally not as rhythmic or focused on the pelvic region as GD behaviors. These actions also tend to occur at times of stress or bedtime.

Management approaches

Management approaches for GD in children involve a multifaceted strategy, including behavioral interventions, pharmacological treatments, educational and environmental adjustments, and family and social support. Each component is vital in addressing the child's and their family's needs [34]. Behavioral interventions are typically the first line of management for GD. Cognitive-behavioral therapy (CBT) techniques can be particularly effective in helping children improve impulse control. CBT enables children to identify triggers for their self-stimulatory behaviors and develop coping strategies to manage these urges [35]. Parent training and behavior modification strategies are also crucial. Parents can be educated about the nature of these behaviors and taught distraction techniques to use during episodes. Positive reinforcement can be employed to reward children for engaging in alternative, non-stimulatory activities, which may help reduce the frequency of gratification episodes [36]. While behavioral approaches are often preferred, pharmacological interventions may be considered in more severe cases. Medications such as escitalopram, an SSRI, can help manage anxiety and improve impulse control, while aripiprazole, an atypical antipsychotic, may provide mood-stabilizing effects [37]. However, it is important to carefully weigh the potential side effects of these medications, such as weight gain, sedation, and gastrointestinal disturbances. Close monitoring is essential to assess the effectiveness and tolerability of pharmacological treatments, ensuring that the benefits outweigh the risks [37].

Educational and environmental modifications can also enhance a child's ability to manage GD. In the classroom, informing teachers about the child’s condition can help create a supportive environment. Strategies such as providing breaks or quiet time can minimize distractions and reduce the likelihood of episodes [1]. Families can implement structured routines at home and engage children in various activities to reduce idle time that may trigger self-stimulatory behaviors. Creating a supportive home environment that encourages open communication about feelings and behaviors fosters a sense of security for the child [1]. Family involvement is critical for the child’s development and effective management of GD. Parents should actively participate in the child’s treatment plan, reinforcing positive behaviors and offering emotional support. Open communication within the family is essential for allowing children to discuss their feelings and behaviors without fear of judgment [38]. Additionally, connecting with other families facing similar challenges through support groups can offer valuable emotional support and practical strategies. By fostering a supportive environment, families can significantly enhance the effectiveness of management approaches for GD, promoting the child's overall well-being and development [38]. Management approaches for GD in childhood are detailed in Table 3.

**Table 3:** Management approaches for GD in childhood CBT, cognitive-behavioral therapy; OCD, obsessive-compulsive disorder; ASD, autism spectrum disorder; GD, gratification disorder

Management Approach	Description	Key Considerations
Parental Education and Reassurance [39]	Educating parents about the benign nature of the disorder and providing reassurance that the behavior is normal and self-limiting.	Emphasize that the condition is typically harmless and resolves with age. Parents should be encouraged to avoid punishment or overreaction.
Distraction Techniques [40]	Engaging the child in stimulating activities or offering toys to divert attention from the repetitive behavior during inactive periods.	Parents can introduce interactive toys, involve the child in physical play, or initiate conversations to distract from the behavior.
Behavioral Modification [41]	Encourage positive reinforcement for alternative behaviors and promote active engagement when gratification behavior is more likely to occur.	Behavior modification can be effective in changing patterns. However, the child should not feel punished for engaging in gratification behavior.
Environmental Changes [42]	Modifying the child’s environment to reduce boredom or extended periods of inactivity, such as increasing outdoor play or interactive activities.	Creating a stimulating and varied environment can decrease the likelihood of episodes. Limit long periods of sedentary activities like screen time.
Monitoring for Co-existing Conditions [43]	Periodically monitoring the child for signs of other neurodevelopmental or psychological conditions (e.g., ASD, OCD) that may require different management strategies.	If behaviors persist or other developmental issues arise, referral to a specialist (e.g., pediatric neurologist and psychologist) may be necessary.
Therapeutic Interventions [44]	If the behavior significantly impacts daily functioning or social development, referral to a child psychologist or therapist may be warranted for further evaluation.	Interventions such as CBT may be considered if the behavior becomes disruptive or concerning to caregivers.
Avoid Punishment or Shame [45]	Caregivers should avoid punishing or shaming the child for engaging in gratification behaviors, as this can lead to anxiety or embarrassment.	Negative responses to the behavior can create emotional distress. Encouraging a calm and supportive approach is critical to management.
Focus on Developmental Milestones [46]	Ensuring the child meets expected developmental milestones in speech, social skills, and motor coordination.	If there are concerns about developmental delays, further investigation into underlying conditions may be needed.
Parental Support Groups [47]	Providing parents with access to support groups or educational materials that help them understand the condition and share experiences with other families.	Support groups can offer emotional reassurance and practical tips from other parents experiencing similar challenges.
Referral to Specialist (If Needed) [23]	In rare cases where the behavior persists or is associated with other symptoms, referral to a pediatric neurologist, psychologist, or developmental specialist may be necessary.	Further evaluation may be required if the child’s severe behavior is disruptive or associated with developmental concerns.

Prognosis and outcomes

GD is generally regarded as a benign condition that typically resolves as children mature, usually by two to three years old. Most children with this disorder follow normal developmental trajectories, and the absence of significant intervention usually does not result in adverse long-term effects [5]. However, some studies suggest that a small percentage of children may develop behavioral issues later, such as characteristics indicative of ADHD. While the immediate impact of GD on academic and social functioning is not extensively documented, the behaviors associated with it can sometimes lead to social stigma or misunderstanding among peers. Thus, while most children will not face long-term challenges, early intervention and support can mitigate potential negative consequences [48]. The prognosis for children with GD is generally excellent, with most experiencing complete resolution of symptoms by toddlerhood. Early recognition of the disorder is important, as it helps alleviate parental concerns and avoids unnecessary medical investigations or interventions. Educating parents about the benign nature of the behavior is essential, as it promotes a supportive environment for the child [5]. In some instances, ongoing support and behavioral therapy may be beneficial, especially if the self-stimulatory behaviors become excessive or distressing for the child. Although there is limited research on the long-term outcomes of GD, early intervention strategies are believed to enhance overall developmental trajectories and may improve academic and social functioning in the long term. Early understanding and intervention can ultimately lead to positive outcomes and facilitate a smoother transition into later childhood [5].

Future directions and research

Despite the growing understanding of GD in children, there are still significant gaps in knowledge that warrant further research. A critical area needing attention is the epidemiology of GD; more extensive studies are required to determine the true prevalence and incidence of this condition across diverse populations and age groups. Gaining insights into how common GD is can help inform healthcare providers and parents, ensuring that those affected receive appropriate support and intervention [1]. The long-term outcomes for children diagnosed with GD are also not well-studied. Research into potential associations between GD and other developmental conditions, such as ADHD, is essential. Longitudinal studies with extended follow-up periods could provide valuable insights into how GD might impact a child's development. Additionally, the pathophysiology underlying GD remains poorly understood. Investigating the neural mechanisms involved in this behavior through neuroimaging and neurophysiological studies may illuminate its origins and help distinguish it from other movement disorders [49]. Cultural influences represent another understudied area in the context of GD. GD's recognition, reporting, and management can vary significantly across different cultural and religious contexts. Research into how these factors affect perceptions of GD could enhance understanding and improve clinical practices in diverse settings [50].

As research into GD progresses, several potential advances in diagnosis and treatment may emerge. One promising direction is the development of standardized diagnostic criteria and tools, which could improve the accuracy and consistency of GD diagnoses across various healthcare settings. Enhanced diagnostic tools would allow healthcare providers to better differentiate GD from other conditions, thereby reducing the risk of misdiagnosis [1]. Additionally, novel neuroimaging techniques, such as functional MRI and positron emission tomography (PET) scans, may help identify specific brain regions or circuits involved in GD. These advancements could lead to improved diagnostic markers and a deeper understanding of the neural correlates of the disorder [51]. On the treatment front, targeted behavioral interventions incorporating mindfulness, distraction, and habit reversal could be explored as potential therapeutic options. Although medication is not typically recommended for GD, further research into the efficacy and safety of selective serotonin reuptake inhibitors (SSRIs) and other psychotropic drugs may be warranted, particularly in cases where behavioral therapy alone proves insufficient [51]. Finally, fostering multidisciplinary collaboration among pediatricians, child psychiatrists, neurologists, and psychologists could lead to the development of comprehensive, evidence-based guidelines for managing GD in children. Such collaboration would ensure that affected children receive holistic care tailored to their needs [51]. Future directions and research in understanding and managing GD in childhood are detailed in Table 4.

**Table 4:** Future directions and research in understanding and managing GD in childhood OCD, obsessive-compulsive disorder; ASD, autism spectrum disorder; OCD, obsessive-compulsive disorder; ADHD, attention-deficit/hyperactivity disorder; GD, gratification disorder

Research Area	Description	Potential Impact
Longitudinal Studies on Prognosis [52]	Conducting long-term studies to track the development of children with GD to determine if the behavior is predictive of other developmental or psychological issues in later childhood or adolescence.	Understanding long-term outcomes can help in early intervention and provide insight into possible developmental correlations.
Neurobiological Underpinnings [53]	Investigating the neurobiological and neurodevelopmental factors associated with GD through neuroimaging and electrophysiological studies.	Discovering neurological pathways involved in the disorder could lead to targeted therapies or a better understanding of its benign nature.
Cultural and Social Influences [54]	Exploring how cultural norms, parenting styles, and environmental factors influence the prevalence and presentation of GD across different populations.	This research could enhance cross-cultural understanding and influence the development of tailored management strategies based on societal norms.
Effectiveness of Behavioral Interventions [55]	Testing various behavioral modification programs, including parent-led strategies, to determine the most effective interventions for managing gratification behaviors.	Identifying effective interventions could improve outcomes for children, reduce parental anxiety, and prevent unnecessary medical evaluations.
Role of Parental Education [1]	Assessing the impact of different educational approaches for parents in managing GD, such as the effectiveness of informational materials versus in-person counseling.	Improved education methods could lead to earlier identification of benign cases and reduce misdiagnosis or over-treatment.
Link to Other Developmental Disorders [56]	Researching possible connections between GD and other developmental or neurological conditions such as ASD, OCD, and ADHD.	Understanding whether GD is linked to other developmental conditions can guide diagnostic practices and treatment plans.
Genetic Predisposition [57]	Investigating whether there is a genetic component to GD by studying familial patterns and genetic markers.	Identifying genetic factors could improve early diagnosis, help differentiate it from other conditions, and lead to more personalized treatments.
Psychological Impact on Children [58]	Studying the potential psychological effects on children who experience parental anxiety or social stigma due to their gratification behaviors.	Reducing stigma and providing mental health support could improve the child's psychological well-being and family dynamics.
Global Prevalence and Epidemiology [59]	Conducting large-scale epidemiological studies to determine the prevalence of GD globally and variations based on geography, race, or socioeconomic status.	Such data can inform healthcare providers and educators about the scope of the disorder and lead to more standardized diagnostic criteria.
Technological Interventions [60]	Exploring the use of technology-based interventions, such as interactive apps or online platforms, to help parents manage GD in their children.	Technological tools could offer new, accessible ways for parents to manage the condition and track behavioral progress over time.

## Conclusions

In conclusion, a thorough understanding of GD is essential for effectively addressing its challenges and supporting affected children. By defining GD and exploring its impact on development and daily functioning, this review highlights the importance of accurate diagnosis and the need for targeted interventions. Examining diagnostic criteria and differential diagnoses underscores the complexity of distinguishing GD from other conditions with similar symptoms. At the same time, the evaluation of management strategies offers practical solutions for clinicians, educators, and parents. Recognizing and addressing GD can significantly enhance a child's ability to develop self-control, achieve academic success, and foster positive social relationships. Continued research and awareness are crucial for advancing our understanding of GD, improving diagnostic tools, and refining treatment approaches. Ultimately, a comprehensive approach to GD will improve the quality of life for affected children and support their long-term developmental outcomes, paving the way for a more adaptive and fulfilling future.
